# Role of Ertugliflozin in the Management of Diabetes Mellitus

**DOI:** 10.7759/cureus.31404

**Published:** 2022-11-12

**Authors:** Manisha Totade, Shilpa A Gaidhane

**Affiliations:** 1 Medicine, Jawaharlal Nehru Medical College, Datta Meghe Institute of Medical Sciences, Wardha, IND

**Keywords:** monotherapy, glycemic control, renal hypoglycemia, cardiovascular effects, ertugliflozin, insulin, diabetes mellitus, sglt2 inhibitor

## Abstract

The novel oral sodium-glucose cotransporter-2 (SGLT2) inhibitor Ertugliflozin (SteglatroTM) is introduced as a monotherapy or in conjunction with another antidiabetic drug regimen for the treatment of type 2 diabetes mellitus (T2DM).Additional safe and efficient treatment options for patients and physicians are of utmost importance as the incidence of T2DM rises. As a standalone therapy or an adjunctive treatment, ertugliflozin seems to be a reliable and safe option.This narrative review seeks to report and analyze ertugliflozin's effectiveness, safety, cardiovascular (CV), and renal outcomes in T2DM. Various combinations of drugs and drug classes have been tried to reduce mortality and comorbidities associated with the use of antidiabetic agents, especially cardiogenic events and renal diseases. With the administration of hypoglycemic drugs like ertugliflozin and the regulation of blood sugar levels, the incidence of therapy-induced hypertension, obesity, and dose-related hypoglycemia has been reduced to a significant extent. Additionally, ertugliflozin prevents hypertension caused by prolonged antidiabetic drug intake, which is advantageous for lowering the chances of end-stage cardiac events in type 2 diabetic patients. As far as the renal safety profile of ertugliflozin is concerned, it has been associated with the maintenance of eGFR (estimated glomerular filtration rate) and a decreased UACR (urine albumin-to-creatinine ratio) in patients with T2DM and coronary artery disease, which reduces the incidence of renal adverse effects due to long-term medication. As a result of common pathophysiological mechanisms, SGLT2 inhibitors represent a feasible therapeutic option and are advantageous for patients with type 1 and type 2 DM in terms of cardiovascular and renal outcomes.

## Introduction and background

Ertugliflozin is a newer drug in the therapy regimen for type 2 diabetes mellitus (T2DM) that aims to decrease the cardiac, microvascular, macrovascular, and renal complications of diabetes mellitus (DM). The U.S. Food and Drug Administration agency (FDA) and other regulatory bodies in Europe and Asia (European Medicines Agency, National Medical Products Administration) have licensed or approved for the oral use of ertugliflozin for regulating blood glucose levels in individuals with T2DM [1-2]. Four medications from this therapeutic family are now accepted in the USA and Europe: ertugliflozin (the most recent Sodium-Glucose Cotransporter-2 (SGLT2) inhibitor), canagliflozin, dapagliflozin, and empagliflozin [3]. The prevalence and complications of T2DM, such as coronary arteriosclerosis, diabetic acidosis, hypoglycemia, etc., are significant global health challenges. Blocking of the sodium-glucose cotransporter-2 can, in an insulin-independent way, enhance glucose excretion from urine and decrease blood glucose levels, The administration of hypoglycemic drugs such as ertugliflozin, along with blood sugar level regulation, has significantly reduced the incidence of therapy-induced hypertension, obesity, and dose-related hypoglycemia [4].

Ertugliflozin has been investigated in both T2DM patients and healthy human volunteers, showing identical pharmacokinetic (PK) profiles in both groups. Ertugliflozin is about 100% bioavailable when given orally and arrives at its highest concentration in less than two hours when fasting. With a 16.6-hour half-life (t1/2) in T2DM patients, ertugliflozin is suitable for once-daily treatment. After four to six days of overdose (OD) treatment, ertugliflozin achieves steady-state concentrations in the body [5]. Regarding the metabolism of ertugliflozin, uridine diphosphate glucuronosyltransferase (UGT) 1A9- and UGT 2B7-mediated O-glucuronidation converts ertugliflozin into two glucuronides that are not pharmacologically active, which also suggests that there is little metabolism mediated by cytochrome P450 [6].

After taking anti-diabetic medication for an extended period, patients with T2DM who were previously free of cardio-renal disorders are at a high risk of developing initial cardio-renal symptoms such as indicators of progressive heart failure and renal malfunctions. These cardiorenal effects are also linked to an increase in the incidence of long-term cardiac complications such as ischemic heart disease and mortality [5]. Chronic hyperglycemia causes this elevated cardiorenal risk, which is aggravated by additional comorbidities including hypertension, dyslipidemia, and obesity. Therefore, there is a need for effective therapies that might aid type 2 diabetic patients in achieving and maintaining plasma glucose level control, along with delaying the beginning and progression of cardiorenal illness. The SGLT2 inhibitors, one of the several families of glucose-lowering drugs, have demonstrated the potential to meet this demand [6-7]. In this narrative review, we plan a detailed study of the drug called ertugliflozin, which is a potent SGLT2 inhibitor, and its role in the prevention of cardiorenal complications of T2DM. No experiments with living subjects, such as humans or animals, were done by any of the writers in this review; instead, it was based on earlier research.

## Review

Cardiovascular effects of ertugliflozin

A significant challenge is treating diabetic complications such as cardiac and vascular pathologies (CVD). It was recently found that SGLT-2 inhibitors decrease the incidence of CVD in diabetic individuals. Therefore, such glucose-lowering medications would be regarded as a significant advancement [8].

In comparison to the control, SGLT2 inhibitor treatment was significantly related to a decreased incidence of atrial arrhythmias and end-cardiac events. Between groups, there were no appreciable differences in incident ventricular arrhythmias or the "cardiac arrest" SCD component [9]. In individuals with T2DM, SGLT2 inhibitors are related to a lower risk of cardiac arrhythmias and failure. Further experiments are needed to determine if the antiarrhythmic action of SGLT2 is drug- or class-specific [10]. Despite this, individuals with T2DM saw a decrease in fatal cardiovascular (CV) events than did controls, and their excess risk remained considerable when compared to non-diabetics [8-11]. Various data analyses that compared the efficiency of DPP-4 inhibitors and newer classes of SGLT-2 inhibitors showed evidence of decreased cardiovascular risk in diabetic patients who were given drugs like ertugliflozin and empagliflozin as compared to linagliptin and saxagliptin. Further research into the events of cardiac diseases and heart failure in non-diabetic patients is required [8].

The cardiovascular effects of empagliflozin, canagliflozin, dapagliflozin, and ertugliflozin in T2DM patients have been monitored in four large-scale trials so far, which are The EMPA-REG OUTCOME experiment (for empagliflozin cardiac results and death rates in T2DM) investigated empagliflozin's potential [7], while the CANVAS PROGRAM [12] (Canagliflozin Cardiovascular Assessment Study) investigated canagliflozin's efficacy [13]. Ertugliflozin did not demonstrate a meaningful difference in the critical 3-point MACE (nonfatal stroke, nonfatal myocardial infarction, and cardiovascular death) result. It was, however, linked to a 30% reduction in hospitalization due to heart failure (HF) [6]. Ertugliflozin acts as a cardioprotective through osmotic diuresis, and plasma volume reduction is thought to increase cardiac preload and afterload, lowering oxygen consumption, which may assist patients with heart failure [15]. The cardiac muscle receives more oxygen thanks to the rise in hematocrit levels. This increase could be brought on by a lowered plasma volume and/or an increase in erythropoietin production [16]. In addition, SGLT2 inhibitors lower body weight and blood pressure, adding to the long list of cardiac and metabolic advantages [17].

The renal outcome of ertugliflozin

It is critical to understand the renal consequences of ertugliflozin. Due to the caloric deficit brought on by the enhanced glucosuria and decreased glucotoxicity, a sequence of metabolic adjustments appears to have begun. This might shield various targeted tissues against oxidative pressure, endothelial malfunction, swelling, and fibrosis in the kidney, arteries, retina, heart, and adipose tissue. Ertugliflozin acts by decreasing sodium and glucose reabsorption from the proximal convoluted tubule and enhancing distal sodium transport to the macula densa; thus, tubule glomerular feedback is restored, leading to a decrease in intra-glomerular pressure, shear stress, hyperfiltration, and albuminuria [18-19], hence it has a reno-protective effect. In addition to improving blood pressure and kidney functions, SGLT2 inhibitors also have additional physiological effects, such as natriuresis [20-21]. Ertugliflozin, in addition to reducing the albumin/creatinine ratio in patients with type 2 diabetes and established diabetic nephropathy, also lowers the risk of kidney composite, which includes a significant decline in renal filtering capacity (doubling of serum creatinine or a sustained 40% decrease in eGFR), renal replacement therapy, and renal death [20-22].

According to cohort research conducted by David Z. I. Cherney et al. [20], the pre-specified exploratory composite renal endpoint analysis was performed in the combined ertugliflozin population by ertugliflozin dose (5 mg and 15 mg). Changes in estimated glomerular filtration rate (eGFR) over time by treatment group are also recorded, as are changes in albuminuria over time and changes in albuminuria status. All analyses were performed on the full population as well as subgroups stratified by baseline renal function. Using the Acute Renal Failure Standard Medical Dictionary for Regulatory Activities Query (SMQ) and two baseline eGFR subgroups, the frequency of adverse events associated with acute kidney failure was assessed [20]. Moreover, it was found that ertugliflozin is a renoprotective agent.

CKD is concomitantly associated with T2DM, which is characterized by persistent albuminuria, a reduction in glomerular filtration rate (GFR), increased blood pressure, and an increased risk of cardiovascular disease [23-24]. SGLT2 inhibitors have a synergistic blood pressure-lowering effect with anti-hypertensive medications. Renal endpoints have been included in several cardiovascular outcomes studies (CVOTs), which has increased the body of data supporting these drugs' potential reno-protective benefits in T2DM patients [25]. More information on the potential therapeutic function of SGLT2 inhibitors in reducing the onset and progression of renal impairment in people with T2DM will emerge from several ongoing dedicated renal outcomes investigations [23].

Ertugliflozin in combination with other drugs

The current gold standard treatment for type 2 diabetes is a combination of antihyperglycemic medications designed to achieve the best glycemic control in the shortest amount of time and in the safest manner possible. Agents should ideally have complementary modes of action that boost glycemic control without unacceptable adverse effects [26].

Ertugliflozin can be used in combination with other drugs, which can act as synergists, resulting in positive outcomes in patients with T2DM [27]. One such drug is sitagliptin; the combination of sitagliptin and ertugliflozin comes with the brand name STEGLUJAN [28]. In a study conducted by Marrs and Anderson et al. [3], ertugliflozin 5 mg plus sitagliptin 100 mg, ertugliflozin 15 mg plus sitagliptin 100 mg, or a placebo were given to 291 patients with T2D who were not adequately controlled with diet and exercise alone (HbA1c 8.0% and 10.5%) for 26 weeks. The primary efficacy endpoint was the change in HbA1c from baseline to week 26 [29]. When compared to a placebo, both ertugliflozin and sitagliptin therapies significantly decreased HbA1c. Similarly, ertugliflozin and sitagliptin substantially decreased free plasma glucose, two-hour post-prandial glucose (PPG), body weight, and systolic blood pressure (SBP) compared to placebo. Overall, the incidence of side effects was minimal, and there was no difference between the treatment and placebo groups in terms of side effects. In a group of patients with uncontrolled T2D, this trial showed that adding ertugliflozin to sitagliptin was efficient and safe [3]. According to the Biopharmaceutical Classification System, ertugliflozin and sitagliptin are class 1 medications [30-31]. When administered together, these two antihyperglycemic medications' distinct but complementary mechanisms of action and favorable safety profiles produce a more potent antihyperglycemic effect than when administered separately [32]. This strategy is anticipated to have clinical advantages for patients with T2DM who cannot control their condition with metformin alone [30].

Metformin combined with ertugliflozin given to individuals with poorly controlled T2DM enhanced glycemic management and decreased body weight and blood pressure but increased genital mycotic infection (GMI) incidence [33-34]. In a double-blind experiment, 621 T2DM patients were randomly assigned to receive ertugliflozin 5 or 15 mg daily, or a placebo, for 26 weeks if they had poor glycemic control (A1C >7.0% and 10.0%) despite taking metformin 1500 mg per day for at least eight weeks before randomization. Compared to a placebo, ertugliflozin dosing led to more considerable A1C reductions. Body weight (reduced by 3 kg with 5 mg, 2.9 kg with 15 mg, and 1.3 kg with placebo; all p 0.001), fasting plasma glucose (FPG), A1C (7%), systolic blood pressure (SBP), and diastolic blood pressure were likewise substantially lower with ertugliflozin at both dosages compared to placebo [33]. Although adverse effects were broadly equal between groups, ertugliflozin caused higher glucose management indicators (GMI) than placebo. Overall, ertugliflozin added to metformin increased the incidence of GMIs while improving glycemic management compared to a placebo [3].

Metformin and sitagliptin, both together, can also be combined with ertugliflozin. Four hundred sixty-four people with T2D who had insufficient glycemic control (A1C >7.0% and 10.5%) despite receiving metformin 1500 mg per day plus sitagliptin 100 mg per day before enrollment were randomly assigned to receive ertugliflozin five or 15 mg per day for 26 weeks or a placebo [35]. The decrease in A1C from the baseline at 26 weeks served as the primary efficacy objective. Compared to a placebo, ertugliflozin dosing led to more considerable A1C reductions. When ertugliflozin five and 15 mg were used instead of a placebo, significant improvements were observed in achieving the A1C objective of 7%. FPG, body weight, and SBP were all significantly lower with ertugliflozin at both doses compared to placebo, and the effects were sustained for 52 weeks (ertugliflozin 5 mg decreased FPG by 1.5 mmol/L, ertugliflozin 15 mg decreased SBP by 1.8 mmol/L, and placebo decreased SBP by 0.1 mmol/L) [3-35]. The GMI rate was more significant in the ertugliflozin group, as in prior studies, and it was more noticeable in women than in men. There was no statistically significant difference between ertugliflozin and placebo in symptomatic hypoglycemia, UTIs, or hypovolemia. Overall, metformin plus sitagliptin showed better glycemic control with a greater rate of GMIs when ertugliflozin was added, which was consistent with other studies [3].

Table 1 shows the advantages and disadvantages of ertugliflozin.

**Table 1:** Advantages & disadvantages of ertugliflozin GFR: glomerular filtration rate; LDL: low-density lipoprotein

Advantages of ertugliflozin	Disadvantages of ertugliflozin
Decrease in blood pressure	Glycosuria
Natriuresis and diuresis without tachycardia	Urinary tract infections: fungal infections
Cardio-protective: decrease in cardiovascular mortality	Risk factor for diabetic ketoacidosis (loss of blood volume)
No hypoglycemia	Increased LDL and osteoporosis (reported but not proven)
Renoprotective: prevents the rate of fall of GFR and reduces hyperfiltration	

## Conclusions

In December 2019, the FDA approved the new SGLT-2 inhibitor ertugliflozin for the treatment of type 2 diabetes mellitus. Its mechanism of action is insulin-independent, which increases glucose excretion in urine and thus protects the patient from side effects like hypoglycemia that occur in patients on oral hypoglycemic agents. When ertugliflozin is used as monotherapy or in conjunction with metformin and sitagliptin, clinical trials have shown improvement in A1C, FPG, and photoplethysmogram (PPG), reduced body weight, and a lowering of SBP in individuals with T2D. Ertugliflozin's most serious side effect is genital mycotic infections. Treatment with ertugliflozin leads to a significant decrease in heart failure and renal outcomes such as chronic kidney disease, renal failure, etc., and a sustained-to-moderate reduction of the composite CV death rate or hospitalization for HF, as well as a moderate reduction of the CV, total mortality, and major adverse cardiac events (MACE). The transition from a standard antihyperglycemic prescription to a substance with a strong indication of cardiorenal protection is underway and nearly complete. So, using ertugliflozin is safe for type 2 diabetes mellitus and patients with cardiorenal complications.
